# Catheter‐Associated *Burkholderia cenocepacia* Infective Endocarditis in a Patient Receiving Maintenance Hemodialysis

**DOI:** 10.1155/crdi/6619649

**Published:** 2026-07-07

**Authors:** Zhong Xhen Khor, Xin Yee Chong, Nor Zanariah Zainol Abidin, Kok Soon Lee

**Affiliations:** ^1^ Department of Internal Medicine, IMU University, Seremban, Negeri Sembilan, Malaysia, imu.edu.my; ^2^ IMU University, Seremban, Negeri Sembilan, Malaysia, imu.edu.my; ^3^ Hospital Tuanku Jaafar, Seremban, Negeri Sembilan, Malaysia; ^4^ Infectious Disease Consultant, Hospital Tuanku Jaafar, Seremban, Negeri Sembilan, Malaysia

**Keywords:** *Burkholderia cenocepacia*, *Burkholderia cepacia* complex, hemodialysis, infective endocarditis, multidrug resistance

## Abstract

*Burkholderia cenocepacia*, a member of the *Burkholderia cepacia* complex (BCC), is an opportunistic, intrinsically multidrug‐resistant Gram‐negative bacillus most commonly associated with nosocomial outbreaks and chronic pulmonary infection in patients with cystic fibrosis. Endocarditis caused by *B. cenocepacia* is extremely rare and has been reported mainly in intravenous drug users or in association with prosthetic valves. We report a fatal case of native mitral valve infective endocarditis caused by *B. cenocepaci*a in a patient with end‐stage renal disease (ESRD) receiving maintenance hemodialysis. A 50‐year‐old woman with diabetes mellitus, hypertension, and ESRD on hemodialysis had recently been treated for *B. cenocepacia* bacteremia, identified by MALDI‐TOF MS, with a 2‐week course of intravenous ceftazidime. During the same admission, thrombosis of her arteriovenous fistula necessitated placement of a central venous dialysis catheter. She represented shortly after discharge with altered mental status. Blood cultures obtained from both peripheral and central sites again yielded Gram‐negative bacilli, with differential time to positivity suggesting a catheter‐related source. The organism was identified as *B. cenocepacia* by MALDI‐TOF mass spectrometry. Transthoracic echocardiography demonstrated a mobile mitral valve vegetation measuring 1.6 × 0.9 cm. Given extensive comorbidities and poor surgical candidacy, care was transitioned to palliation and the patient died during hospitalization. This case highlights the potential for *B. cenocepacia* to cause invasive endocardial infection in hemodialysis patients with indwelling central venous catheters.

**Learning Points**
• *Burkholderia cenocepacia* endocarditis is extremely rare but should be considered in patients with persistent or recurrent *B. cenocepacia* or BCC bacteremia.
• Recurrent *B. cenocepacia* or BCC bacteremia should prompt early echocardiographic evaluation to exclude infective endocarditis.
• The intrinsic multidrug resistance and biofilm‐forming capacity of *B. cenocepacia* may contribute to persistent bacteremia and treatment challenges, particularly in hemodialysis patients with indwelling dialysis catheters.
• This case highlights the importance of early source control and careful antimicrobial selection in managing catheter‐associated *Burkholderia* infections.

## 1. Introduction

*Burkholderia cenocepacia*, a member of the *Burkholderia cepacia* complex (BCC), is an opportunistic nonfermenting Gram‐negative bacillus widely found in soil and water. It is intrinsically resistant to multiple antimicrobial agents and disinfectants, allowing it to persist in healthcare environments and occasionally cause nosocomial outbreaks. BCC organisms are mostly associated with chronic pulmonary infection in cystic fibrosis and hospital‐acquired bacteremia in critically ill patients without cystic fibrosis [1, 2].

Infective endocarditis caused by BCC organisms is rare and has been reported mainly in intravenous drug users or in association with prosthetic valves or intracardiac foreign material. Because of its rarity and intrinsic multidrug resistance, optimal antimicrobial therapy and treatment duration remain poorly defined [3].

We report a fatal case of native mitral valve infective endocarditis caused by *Burkholderia cenocepacia* in a patient with end‐stage renal disease (ESRD) on maintenance hemodialysis, highlighting a potentially under‐recognized risk population and the therapeutic challenges posed by this organism.

## 2. Case Presentation

A 50‐year‐old woman with diabetes mellitus, hypertension, and ESRD on maintenance hemodialysis was admitted after being found lethargic and poorly responsive. She had recently been treated for *Burkholderia cenocepacia* bacteremia, identified by MALDI‐TOF MS with a log score of 2.20, with a 2‐week course of intravenous ceftazidime during a prior hospitalization.

During that admission, she developed acute right‐sided weakness and CT imaging confirmed a left middle cerebral artery infarct. Her arteriovenous fistula subsequently thrombosed, requiring insertion of a left internal jugular dialysis catheter for ongoing vascular access. She also developed progressive discoloration of the left lower limb attributed to vasopressor use, and CT angiography demonstrated significant peripheral arterial disease with approximately 60% stenosis of the left common iliac artery and 90% stenosis of the left common femoral artery.

On representation, she was hemodynamically stable but lethargic and poorly cooperative. She responded only briefly to verbal stimuli. Neurological examination was limited, although right‐sided weakness was noted, in keeping with the recent left middle cerebral artery infarct from the preceding admission. A complete neurological examination could not be performed. There were no documented peripheral stigmata of infective endocarditis. Cardiovascular examination revealed a loud holosystolic murmur radiating to the axilla, which had not been documented previously. Laboratory investigations showed leukocytosis, anemia, thrombocytopenia, elevated inflammatory markers, and biochemical features of ESRD, with WBC 18.6–23.4 × 10^9^/L, hemoglobin 10.5–7.2 g/dL, platelets 125–104 × 10^9^/L, CRP 28 mg/L, urea 21.8–27.1 mmol/L, and creatinine 844–937 μmol/L. Repeat CT brain demonstrated expected evolution of the prior infarct without new hemorrhage.

Blood cultures were obtained from both peripheral sites and the indwelling dialysis catheter. Empiric intravenous amoxicillin–clavulanate was initiated for suspected hospital‐acquired infection. Two days later, both central and peripheral blood cultures grew Gram‐negative bacilli, with the catheter‐drawn culture becoming positive more than 2 hours earlier than the peripheral culture, supporting a catheter‐related bloodstream infection. Both catheter‐drawn and peripheral blood culture bottles contained similar blood volumes of 10 mL.

**FIGURE 1:**
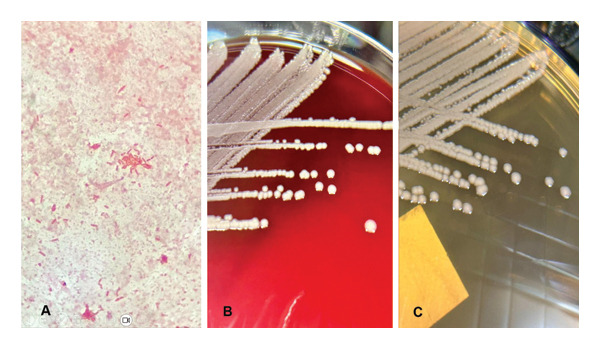
(A) Gram‐negative bacillus on Gram stain. (B) Isolates on blood agar appear smooth, convex, mucoid, nonhemolytic greyish white to pale yellow colonies after 18 h of incubation at 35°C. (C) Non‐lactose‐fermenting colonies on MacConkey agar.

The organism produced smooth mucoid colonies on blood agar and non‐lactose‐fermenting colonies on MacConkey agar (Figure 1). Identification by matrix‐assisted laser desorption ionization‐time of flight mass spectrometry (MALDI‐TOF MS) confirmed *Burkholderia cenocepacia* with a log score of 2.37 indicating high confidence identification. Molecular characterization was not performed, as this was not part of routine laboratory workflow.

Antimicrobial susceptibility testing was performed for ceftazidime, meropenem, and trimethoprim–sulfamethoxazole, with MIC values of 4, 2, and 0.38 μg/mL, respectively. However, clinical interpretive breakpoints for the BCC have been removed from the CLSI M100 guidelines due to the lack of validated and reproducible testing methodologies [4].

Transthoracic echocardiography demonstrated a mobile vegetation measuring 1.6 × 0.9 cm on the mitral valve, consistent with infective endocarditis. Left ventricular systolic function was preserved with an estimated ejection fraction of 60%. No alternative septic focus was identified.

Given the patient’s multiple comorbidities and poor surgical candidacy, valve surgery was not pursued. After discussions with family members, care was transitioned to comfort‐focused management. The dialysis catheter was removed, and the patient died during the same hospitalization.

## 3. Discussion

Endocarditis caused by BCC organisms remains extremely rare. Most reported cases have occurred in intravenous drug users or in patients with prosthetic cardiac valves, structural heart disease, or intracardiac foreign material [5–18]. In contrast, reports involving patients receiving maintenance hemodialysis remain exceedingly limited. To our knowledge, native‐valve infective endocarditis caused by *Burkholderia cenocepacia* in a patient receiving chronic hemodialysis has rarely, if ever, been described. The novelty of this case lies not merely in *Burkholderia cenocepacia* bacteremia in a dialysis patient but in recurrent *Burkholderia cenocepacia* bloodstream infection complicated by native mitral valve infective endocarditis in the setting of hemodialysis vascular access.

A literature search was performed using PubMed and Google Scholar from database inception to June 2026. Search terms included “*Burkholderia cenocepacia* endocarditis,” “*Burkholderia cepacia* endocarditis,” “*Burkholderia cepacia* complex endocarditis,” “native valve endocarditis,” “hemodialysis,” “dialysis,” “catheter‐related bloodstream infection,” and “bacteremia.” Reference lists of relevant case reports and reviews were also screened. Cases were considered relevant if they described BCC bacteremia or infective endocarditis, particularly in dialysis, catheter‐related, nosocomial, prosthetic‐valve, native‐valve, or immunocompromised settings (see Table 1).

**TABLE 1:** Summary of reported infective endocarditis cases caused by *Burkholderia cepacia* complex organisms.

Author (Year)	Age	Sex	Co morbidity	Valve involved	Vegetation size (cm)	Prosthetic valve	Antimicrobial	Surgery	Outcome
Aggarwal (2005)	58	F	Not specified	Multiple	NR	Yes	TMP–SMX monotherapy	No	NR
Saaraswat (2009)	47	M	Recurrent IE	Aortic	NR	Yes	Antibacterial + antifungal (dual pathogen)	No	Recovered
Ki (2011)	77	F	None	Mitral	1.3 cm	No	Ceftriaxone + aminoglycoside, then ceftazidime (Day 7)	No	Recovered
Williamson (2011)	44	M	IVDU	Tricuspid	NR	No	TMP–SMX combination	No	Recovered
Balaji (2013)	28	F	Rheumatic HD	Mitral	NR	Yes	TMP–SMX–based regimen	MVR	Recovered
Russo (2017)	78	F	Prior stroke	Mitral	NR	Yes	Ceftriaxone and clarithromycin were replaced by cefepime (2 g every 8 h).	MVR	Recovered
Sabir (2018)	30	M	Rheumatic heart disease, prior IE	Aortic + Mitral	1.4 × 1.7 cm	Yes	TMP–SMX combination	AVR + MVR	Recovered
Dellalana (2019)	38	M	IVDU	Mitral	3.2 × 2.2 cm	Yes	TMP–SMX combination	MVR	Relapse
Nnaoma (2019)	32	M	IVDU, prior IE	Mitral	NR	Yes	Broad‐spectrum combination	MVR	Recovered
Moy (2022)	56	M	Bicuspid aortic valve, aortic aneurysm	Aortic	0.7 × 0.6 cm	Yes	Multidrug salvage regimen	No	Died
Almubarak (2024)	54	F	Hypertension	Aortic	3.0 × 2.1 cm	No	Broad‐spectrum β‐lactam	AVR	Recovered
Pepe (2024)	76	F	Long‐standing murmur	Mitral	0.9 × 0.9 cm; 2.4 × 1.3 cm abscess	No	Not specified	No	Died
Gonzalez (2024)	37	M	Prior IE, MVR	Mitral	NR	Yes	Combination β‐lactam regimen	MVR + TVR	Recovered
Mehmood (2025)	55	M	Aortic stenosis	Aortic	NR	Yes	TMP–SMX combination	No	Recovered
Present case (2025)	**50**	**F**	**ESRD, hemodialysis catheter**	**Mitral**	**1.6 × 0.9 cm**	**No**	**Ceftazidime-based therapy, then meropenem**	**No**	**Died**

*Note:* TMP–SMX: trimethoprim–sulfamethoxazole. The bold values represent the index case in this report.

Abbreviations: AVR, aortic valve replacement; ESRD, end‐stage renal disease; IE, infective endocarditis; IVDU, intravenous drug user; MVR, mitral valve replacement; NR, not reported; TVR, tricuspid valve replacement.

Our case suggests that central venous dialysis catheters may represent an additional risk factor for BCC endocarditis. BCC bacteremia is commonly hospital acquired and frequently associated with contaminated solutions or indwelling vascular devices [1, 3, 19]. BCC organisms are able to persist in healthcare environments because of their intrinsic resistance to multiple antimicrobial agents and disinfectants [2]. However, progression from bacteremia to infective endocarditis appears to occur only sporadically.

In the present case, the first admission was retrospectively notable. The patient had *Burkholderia cenocepacia* bacteremia, ischemic stroke, and subsequent thrombosis of her arteriovenous fistula. Echocardiography was not performed during that admission because there was no documented murmur or peripheral stigmata of infective endocarditis, and the bacteremia was treated as uncomplicated *Burkholderia cenocepacia* bacteremia with a 2‐week course of ceftazidime. Although the stroke was not clinically attributed to septic embolism and the peripheral arterial disease was considered chronic, infective endocarditis cannot be definitively excluded retrospectively. In view of the subsequent diagnosis of mitral valve vegetation, IE may already have been present or evolving during the first hospitalization. This case, therefore, highlights the importance of considering IE in hemodialysis patients with BCC bacteremia, particularly when bacteremia recurs or is accompanied by vascular access thrombosis, neurological events, or unexplained clinical deterioration.

Microbiological clearance after the first admission was not documented as repeat blood cultures were not obtained after completion of antibiotics. This is a limitation of the case. The recurrence of *Burkholderia cenocepacia* bacteremia shortly after discharge raises the possibility of persistent infection, relapse, or ongoing catheter‐related seeding. In hemodialysis patients, the documentation of blood‐culture clearance is especially important because vascular access devices may serve as persistent reservoirs for infection.

Both the initial and subsequent isolates were identified as *Burkholderia cenocepacia* by MALDI‐TOF MS, with log scores of 2.20 and 2.37, respectively. This supports species‐level recurrence of the same organism. However, molecular typing or whole‐genome sequencing was not performed; therefore, strain‐level identity between the two episodes could not be definitively proven. Nevertheless, the close temporal relationship, recurrence with the same species, and compatible clinical course suggest that the two episodes were likely related.

The source of infection was most plausibly healthcare associated, with the dialysis catheter or vascular access pathway as the leading consideration. Blood cultures from both the peripheral site and dialysis catheter yielded the same organism, with the catheter‐drawn culture becoming positive more than 2 hours earlier than the peripheral culture despite similar blood volumes [20]. This pattern supports a catheter‐related bloodstream infection. Environmental sampling was not performed, so contamination from healthcare water sources, dialysis‐related handling, or other nosocomial reservoirs could not be confirmed.

Review of published cases reveals several recurring features. Mitral valve involvement appears most common in nonintravenous drug users, whereas tricuspid valve infection predominates among intravenous drug users. Many cases occur in the presence of prosthetic material or intravascular devices, suggesting that endothelial disruption or foreign surfaces facilitate bacterial adherence.

Management of BCC infections is complicated by intrinsic antimicrobial resistance. Multiple mechanisms contribute to this phenotype, including inducible β‐lactamases, reduced outer membrane permeability, efflux pumps, and biofilm formation [2]. These mechanisms contribute to treatment failure and persistent bacteremia, particularly when intravascular devices remain in situ [1, 3, 19].

Recent surveillance and outbreak investigations have also documented increasing resistance or intermediate susceptibility to commonly used agents such as ceftazidime, TMP–SMX, and carbapenems, further narrowing therapeutic options [19]. Early removal of infected vascular catheters is, therefore, critical in achieving source control and improving clinical outcomes in BCC bacteremia and related infections [1, 3, 19].

Combination antimicrobial therapy has been explored in experimental settings. In vitro studies demonstrate potential synergistic activity between β‐lactams and aminoglycosides or carbapenems combined with biofilm‐disrupting agents such as N‐acetylcysteine [21]. However, clinical outcome data supporting these strategies remain limited.

Therapeutic options in ESRD patients are further restricted because aminoglycosides and polymyxins carry significant nephrotoxicity and neurotoxicity. Consequently, treatment is often limited to β‐lactam‐based regimens or trimethoprim–sulfamethoxazole, which may themselves exhibit reduced efficacy in the setting of emerging resistance [19, 21]. In patients with endocarditis, antimicrobial therapy alone may be insufficient when there is a large vegetation, persistent bacteremia, embolic complications, or poor source control. However, surgical management may not be feasible in patients with advanced comorbidity, frailty, or poor functional status, as illustrated in this case.

## 4. Conclusion

This case highlights several practical lessons. First, *Burkholderia cenocepacia* bacteremia in a hemodialysis patient should not automatically be treated as uncomplicated bacteremia, particularly when associated with recurrent positive cultures, neurological events, vascular access thrombosis, or unexplained clinical deterioration. Second, repeat blood cultures should be obtained to document clearance. Third, early echocardiography should be considered even in the absence of classical peripheral stigmata or an audible murmur if there are embolic or vascular‐access complications. Finally, catheter‐related infection should be assessed rigorously, including the documentation of blood‐culture source, blood volume, differential time to positivity, and catheter‐tip culture where available.

## Author Contributions

Zhong Xhen Khor conceptualized the study and collected the data. Zhong Xhen Khor and Xin Yee Chong drafted the article. Nor Zanariah Zainol Abidin is responsible for the images and the interpretation of the data. Zhong Xhen Khor, Xin Yee Chong, Kok Soon Lee, and Nor Zanariah Zainol Abidin revised the manuscript and approved the final copy.

Zhong Xhen Khor is the guarantor of the manuscript and takes responsibility for the integrity of the work as a whole.

## Funding

No funding was received for this work.

## Ethics Statement

Written informed consent from the patient could not be obtained because the patient was deceased. Consent from next of kin was not obtained. The case has been sufficiently anonymized in accordance with ICMJE recommendations, and no identifying patient information is included. Institutional approval for publication was obtained from the National Institutes of Health, Ministry of Health Malaysia, in accordance with local publication governance requirements.

## Conflicts of Interest

The authors declare no conflicts of interest.

## Data Availability

No datasets were generated during the current study.
